# Perceptions of the acceptability and feasibility of reducing occupational sitting: review and thematic synthesis

**DOI:** 10.1186/s12966-018-0718-9

**Published:** 2018-09-18

**Authors:** Nyssa T. Hadgraft, Charlotte L. Brakenridge, David W. Dunstan, Neville Owen, Genevieve N. Healy, Sheleigh P. Lawler

**Affiliations:** 1 Centre for Urban Transitions, Swinburne University of Technology, Melbourne, VIC Australia; 2 Baker Heart and Diabetes Institute, Melbourne, VIC Australia; 3 School of Public Health, The University of Queensland, Herston Rd, Herston, Brisbane, QLD 4006 Australia; 4 RECOVER Injury Research Centre, The University of Queensland, Brisbane, QLD Australia; 5 Faculty of Medicine, Nursing and Health Sciences, Monash University, Melbourne, VIC Australia; 6 Institute of Physical Activity and Nutrition Research, School of Exercise and Nutrition Sciences, Deakin University, Melbourne, VIC Australia; 7 Mary MacKillop Institute of Health Research, Australian Catholic University, Melbourne, VIC Australia; 8 School of Sport Science, Exercise and Health, The University of Western Australia, Perth, WA Australia; 9 Melbourne School of Population and Global Health, The University of Melbourne, Melbourne, VIC Australia; 10 Curtin University, School of Physiotherapy and Exercise Science, Perth, WA Australia

**Keywords:** Qualitative, Workplace, Sedentary behaviour, Sitting, Thematic synthesis

## Abstract

**Background:**

Reducing workplace sedentary behaviour (sitting) is a topic of contemporary public health and occupational health interest. Understanding workers’ perspectives on the feasibility and acceptability of strategies, and barriers and facilitators to reducing workplace sitting time, can help inform the design and implementation of targeted interventions. The aim of this qualitative synthesis was to identify and synthesise the evidence on factors perceived to influence the acceptability and feasibility of reducing sitting at work, without, and with, an associated intervention component.

**Methods:**

A systematic search of the peer-reviewed literature was conducted across multiple databases in October 2017 to identify studies with a qualitative component relating to reducing workplace sitting time. Relevant data were extracted and imported into NVivo, and analysed by three of the authors by coding the results sections of papers line-by-line, with codes organised into sub-themes and then into overarching themes. Studies with and without an associated intervention were analysed separately.

**Results:**

Thirty-two studies met the inclusion criteria, 22 of which had collected qualitative data during and/or following a workplace intervention. Sample sizes ranged from five through to 71 participants. Studies predominately involved desk-based workers (28/32) and were most frequently conducted in Australia, USA or the United Kingdom (26/32). Similar themes were identified across non-intervention and intervention studies, particularly relating to barriers and facilitators to reducing workplace sitting. Predominately, work and social environment attributes were identified as barriers/facilitators, with desk-based work and work pressures influencing the perceived feasibility of reducing sitting, particularly for low-cost interventions. Support from co-workers and managers was considered a key facilitator to reducing sitting, while social norms that discouraged movement were a prominent barrier. Across all studies, some consistent perceptions of benefits to reducing sitting were identified, including improved physical health, enhanced emotional well-being and associated work-related benefits.

**Conclusion:**

Common barriers and facilitators to reducing workplace sitting time were identified across the literature, most prominently involving the social environment and job-related demands. These findings can inform the design and implementation of workplace sitting reduction strategies. To increase the generalisability of findings, further research is needed in a more diverse range of countries and industries.

**Electronic supplementary material:**

The online version of this article (10.1186/s12966-018-0718-9) contains supplementary material, which is available to authorized users.

## Background

Technological and societal changes over recent decades have led to a decline in manual-based occupations and a rise in professional and service-related occupations [1]. Associated with the increase in desk-based jobs, many adults now have minimal need or opportunity to perform light or moderate intensity physical activities during working hours. Instead, the majority of work hours can be spent sitting, often for prolonged, unbroken periods of time [2, 3]. High levels of sitting time have been shown to be associated with increased risk of developing type 2 diabetes and cardiovascular disease, and with premature mortality [4, 5]. As such, the workplace has become a priority setting for addressing this chronic disease risk factor [6].

In recent years, a number of systematic reviews have been published on the outcomes of trials examining the effectiveness of approaches to reducing workplace sitting time [7–11]. A common approach has been to alter the physical workplace environment through the provision of activity-permissive workstations, such as sit-stand desks [8]. Overall, environmental-based and multi-component intervention approaches (incorporating individual and/or organisational-level elements alongside environmental changes) have led to the greatest reductions in workplace sitting time [7, 9]. Sit-stand workstations have been more widely available in recent years, but cost implications may still be a barrier to widespread uptake [12, 13].

A limitation of these effectiveness studies is they often provide limited insight into the contextual factors that may influence the extent of behavioural change during such initiatives. To inform the real world implementation of approaches to reduce workplace sitting it is important to better understand workers’ perceptions of the conditions that promote sedentary behaviour in the workplace, and how they understand the factors that may act as barriers to reducing sitting in the context of sitting reduction interventions. Qualitative research, which seeks to explore questions relating to how or why a phenomenon occurs [14], can be informative for supplementing findings gained through quantitative methods (e.g. how much behaviour or health-related change has occurred), or for understanding people’s experiences and perceptions about particular phenomena [14]. When conducted rigorously with critical analysis of the data, testing of assumptions and alternative explanations for findings, qualitative research can provide evidence about participants’ experience or interpretation of a particular phenomenon of interest [15].

Although a body of qualitative evidence relating to workplace sitting interventions has developed, there are no published reviews or syntheses that identify common themes across studies, interventions or populations. Such a synthesis could assist with understanding consistent conditions and factors perceived to influence workplace sitting time; and, could inform the design and refinement of specific future intervention trials and practical initiatives. In addition, identifying gaps in the literature—such as identifying underrepresented occupations or industry sectors—could assist with prioritising future research.

As a basis of informing the translation of such research findings into practice, we aimed to identify and synthesise the qualitative evidence on factors perceived to influence the acceptability and feasibility of reducing sitting at work. Using thematic synthesis methods to summarise the evidence, this review included studies that explored the perceptions of workers, managers, and other relevant stakeholders in specific workplaces (e.g. occupational health and safety professionals), in relation to reducing workplace sitting.

## Methods

### Literature search and selection criteria

A systematic search of the peer-reviewed literature was conducted in the databases: PubMed, Web of Science, Embase, PsycInfo, Business Source Complete and CINAHL on 11 October 2017. Terms relating to workplace, sitting/sedentary behaviour and qualitative methodology were used to search the databases. An example search strategy used for one of the databases is included in Additional file 1. To be included in the review, the following inclusion criteria had to be met: a) sample included working adults in a workplace setting; b) some qualitative data component was included (such as semi-structured interviews, focus groups, open-ended survey questions); c) study reported workers’ perceptions on the feasibility or acceptability of reducing their sitting time at work or their experience of a workplace sitting time targeted intervention; d) in English language; e) peer-reviewed paper.

Studies were excluded if they focused only on increasing moderate-vigorous physical activity, without specifically referring to reducing sedentary behaviour or sitting time. This included interventions focused on encouraging active transport use, fitness classes/gym use during work breaks, or increasing step counts, without an emphasis on reducing sitting across the workday. Studies were also excluded if they focused solely on the perspectives of external agents to the workplace, without the inclusion of individual workers’ perspectives; and, if the study existed only in abstract form. The protocol for this systematic review was retrospectively registered on PROSPERO on 8 December 2017 (CRD42017081880).

Unlike quantitative systematic reviews and meta-analyses, which aim to identify all relevant papers on a particular topic, the focus of syntheses of qualitative research is often to achieve ‘conceptual saturation’, or seeking variability in the themes identified [16]. However, as this may be difficult to achieve in practice, the inclusive systematic approach outlined above was considered the most appropriate method to find all relevant studies.

Two authors (NH and CB) ran the database searches and conducted the screening process. Search results from each database search were first exported to Endnote and duplicates removed. The two authors then conducted the screening process independently. Titles and abstracts of results were screened against the inclusion criteria, and articles clearly not meeting the inclusion criteria were excluded. The full text of the remaining articles were then obtained for screening against the inclusion and exclusion criteria. Following this process, consensus was reached (after consultation with a third author, SL or GH, where necessary) regarding the final list of articles to be included. Reference lists and the authors’ personal reference libraries were also searched for possible additional papers.

**Fig. 1:**
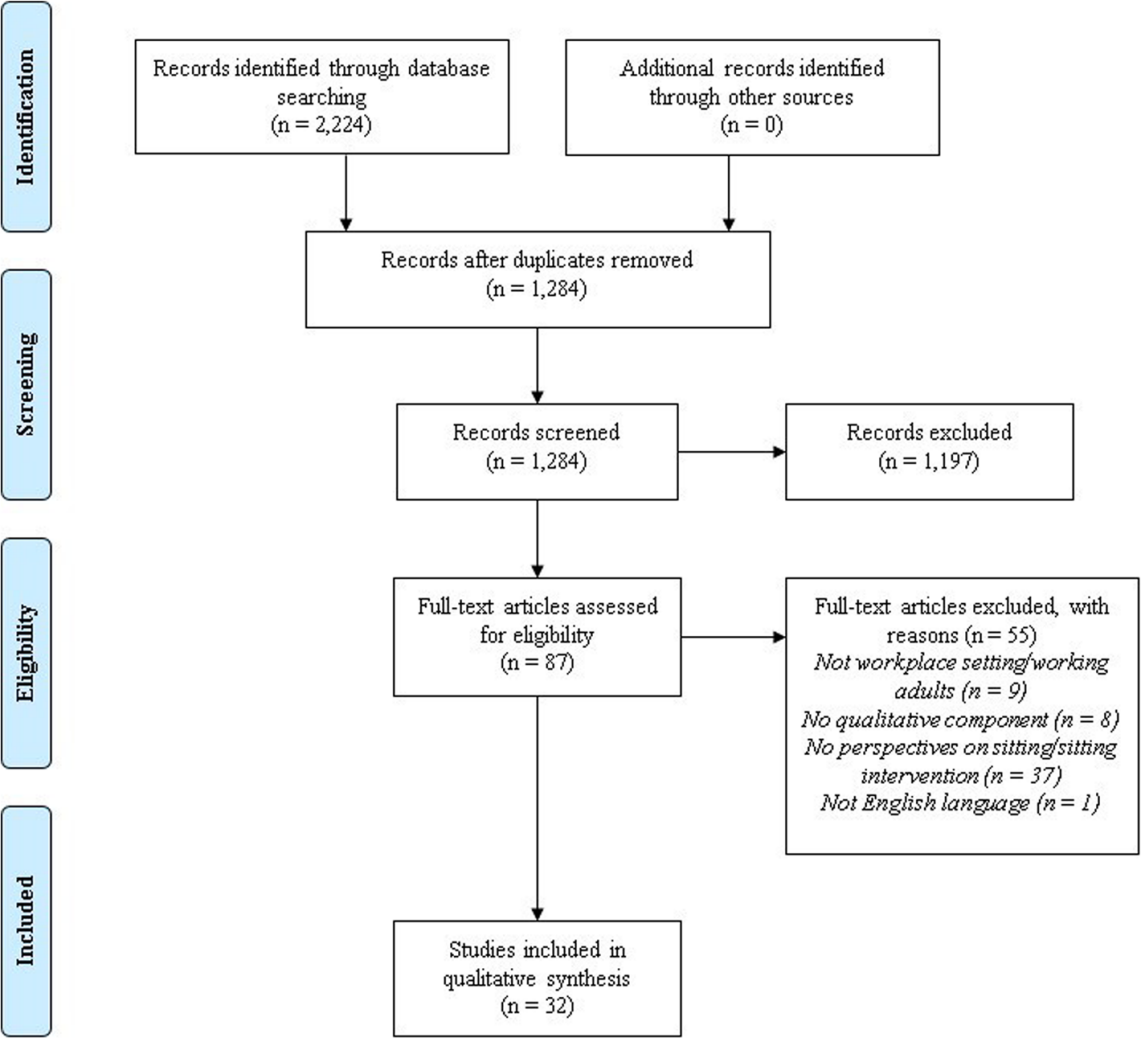
PRISMA flow diagram of study selection

A PRISMA [17] flow diagram of the search process is included in Fig. 1. After duplicates were removed and records screened for relevance based on the title and abstract, 87 articles were assessed in full text for eligibility. After inclusion criteria were applied, 32 studies were selected for inclusion in the qualitative synthesis.

Quality of the included studies was appraised using a modified version of the Critical Appraisal Skills Programme (CASP) qualitative checklist [18], with an additional question added relating to discussion of the limitations of the findings. Appraisals were conducted by NH and either CB or GH, with any discrepancies resolved through consensus. Assessment criteria and a summary of ratings across studies are presented in Table 1. A decision was made not to exclude any papers on the basis of quality as qualitative research is characterised by a diversity of methodological approaches, and studies can sometimes present rich and insightful accounts of the data, yet be limited by poor reporting of their methods [19].

**Table 1:** Summary of quality appraisals across studies

Items assessed	Number of studies		
	Yes	No	Can’t tell
1. Was there a clear statement of the aims of the research?	28/32 87.5%	4/32 12.5%	–
2. Is a qualitative methodology appropriate?	32/32 100%	–	–
3. Was the research design appropriate to address the aims of the research?	13/32 40.6%	4/32 12.5%	15/32 46.9%
4. Was the recruitment strategy appropriate to the aims of the research?	10/32 31.3%	5/32 15.6%	17/32 53.1%
5. Was the data collected in a way that addressed the research issue?	15/32 46.9%	2/32 6.3%	15/32 46.9%
6. Has the relationship between researcher and participants been adequately considered?	3/32 9.4%	29/32 90.6%	–
7. Have ethical issues been taken into consideration?	24/32 75.0%	–	8/32 25.0%
8. Was the data analysis sufficiently rigorous?	10/32 31.3%	14/32 43.8%	8/32 25.0%
9 Is there a clear statement of findings?	17/32 53.1%	14/32 43.8%	1/32 3.1%
10 How valuable is the research?	32/32 100.0%	–	–
11 Limitations discussed / credibility of the findings	16/32 50.0%	16/32 50.0%	–

### Data extraction and analysis

Thematic synthesis was used to analyse the study findings. Relevant data analysed were all text in the ‘results’ sections of included studies, including participant quotes and authors’ analysis. These data were extracted verbatim into NVivo for each study. Studies were characterised as ‘non-intervention studies’, which explored workers’ perspectives on workplace sitting time in the absence of an intervention, and ‘intervention studies’, which generally sought to obtain workers’ perspectives of their experience of participating in an intervention to reduce workplace sitting. As per the process described by Thomas and Harden [16], the first process of analysis involved line-by-line coding of the study findings. To develop the initial coding structure, three authors (CB, SL, NH) separately coded five papers, chosen to incorporate a mix of intervention and non-intervention studies. Through discussion, the initial codes were reviewed and organised into sub-themes, with separate themes identified for intervention and non-intervention studies. This framework was used as the basis for coding the remaining papers. Additional codes and sub-themes were added where necessary, and discussed between the three authors for consistency. After coding was completed, any discrepancies were resolved through consensus between the three authors and final overarching themes were agreed upon. Where common codes and sub-themes were identified across intervention/non-intervention studies, similarities and differences were presented in the results section. Each theme is discussed in detail; including quotes from individual studies were relevant.

## Results

### Study characteristics

Characteristics of the included studies are presented in Table 2. Of the 32 studies, 20 collected qualitative data during and/or following a workplace intervention, 10 did not involve an intervention and two had both (qualitative component before and after an intervention). Of the 22 reporting on interventions, eight involved activity permissive workstations (five sit-stand workstations, two treadmill desks, one bicycle desk), two were multi-component interventions (sit-stand workstations plus individual and organisational-level strategies), four involved walking meetings or routes, three were pedometer-based interventions, and the remainder used a variety of other strategies (generally low-cost) to encourage less sitting and/or more movement (computer prompts [one]; multiple strategies [two], physical activity during breaks [one], mobile app [one]). Sixteen were researcher-led interventions, two were participatory (organisation-led) and two were mixed (components of researcher-led and organisation-led).

**Table 2:** Characteristics of studies included in the review

Study	Location	Sample size (% women)	Intervention sample size	Sector; job types	Intervention description (duration)	Data collection method	Time point of data collection	Data analysis method	Researcher/participatory driven intervention
Non-intervention studies									
De Cocker et al., 2015 [13]	Belgium	55 (56%)	N/A	Manufacturing, port company, human resources; administrative and executives	N/A	Focus groups	N/A	Inductive content analysis	–
Dobson et al., 2013 [21]	USA	20 (“Majority of participants were men”)	N/A	Emergency services; firefighters, engineers, captains and battalion chiefs	N/A	Focus groups	N/A	Grounded theory	–
Flint et al., 2017 [45]	UK	21 (48%)	N/A	Office work; desk-based workers	N/A	Focus groups	N/A	Framework analysis	–
George et al., 2014 [46]	Australia	15 (0%)	N/A	University; faculty and professional staff	N/A	Focus groups	N/A	Thematic analysis	–
Gilson et al., 2011 [28]	Australia	24 (92%)	N/A	Government; service centre employees and team leaders	N/A	Focus groups	N/A	General qualitative analysis	–
Hadgraft et al., 2016 [12]	Australia	20 (50%)	N/A	Not for profit, retail, IT; desk-based, mix of job tasks, 50% management	N/A	Semi-structured interviews, workers, management and OHS staff	N/A	Thematic analysis	–
Loffler et al., 2015 [47]	Germany	10 (20%)	N/A	University and service sector; office employees	N/A	Contextual inquiry interviews	N/A	Contextual inquiry	–
McGuckin et al., 2017 [48]	Australia	12 (92%)	N/A	University; administrative staff	N/A	Focus groups	N/A	Thematic analysis	–
Waters et al., 2016 [27]	Singapore	40 (60%)	N/A	University; non-academic desk based	N/A	Focus groups	N/A	Thematic analysis	–
Wong et al., 2014 [22]	Australia	Drivers: 26 (0%); Managers: 6 (gender NR)	N/A	Transport; bus drivers	N/A	Semi-structured interviews	N/A	Generic qualitative analysis	–
Intervention studies									
Bort-Roig et al., 2014 [37]	Spain	12 (50%)	88	University; academics and administrators	Web-based strategies + pedometer to encourage incidental movement and walking (19 weeks)	Semi-structured interviews	After weeks 4, 8 and 19 (end of intervention)	Inductive open coding	Researcher
Brakenridge et al., 2017 [38]	Australia	Interviews: 50 (46%); focus groups: 21 (38%)	153	Property and infrastructure; desk-based, managerial.	Organisational-support strategies, delivered by a workplace champion. One group also received an activity tracker (12 months)	Semi-structured interviews; focus groups	Interviews: 6–10 months; focus groups: 16 months	Thematic analysis	Participatory
Chau et al., 2014 [29]	Australia	42 (86%)	42	Non-government health; desk-based workers	Sit-stand workstation (4 weeks)	Focus groups	After conclusion of the intervention	Thematic analysis	Researcher
Chau et al., 2016 [49]	Australia	39 (72%)	Not reported	Emergency services; call centre workers	Awareness raising; time lights, emails, posters (10.7 weeks)	Open-ended questions, emails, field notes	Surveys: 5 and 10 months; researcher field notes during the intervention	General qualitative analysis	Researcher
Cifuentes et al., 2015 [20]	USA	5 (100%)	5	University; desk-based workers	Treadmill desk & 2 visits by ergonomists (6 months)	Individual and group interviews	Individual interviews and group interviews: at least monthly during intervention	Unclear	Researcher
Cooley et al., 2014 [35]	Australia	15 (73%)	20	Police; majority non-operational staff, some operational	Computer prompt software (13 weeks)	Semi-structured interviews	After conclusion of the intervention	Typological analytical approach	Researcher
Dutta et al., 2015 [30]	USA	25^a^ (64%)	28	Manufacturing; employees, supervisors, non-participants	Sit-stand workstation & weekly email reminders, anti-fatigue mats, ergonomic evaluation (4 weeks)	Interviews and focus groups	Midpoint and after conclusion of the intervention	Grounded theory	Researcher
Gilson et al., 2008 [50]	UK	15 (87%)	42	University; academics and administrative staff	Intervention promoting walking routes, or walking while working (10 weeks)	Semi-structured interviews	After conclusion of the intervention	Thematic analysis	Researcher
Graves et al., 2015 [31]	UK	7 (100%)	23	University; predominately administrative staff	Sit-stand workstation & basic ergonomic information on use (8 weeks)	Focus groups	After conclusion of the intervention	Content analysis	Researcher
Grunseit et al., 2013 [32]	Australia	12 (gender NR)	31	Government; office workers	Sit-stand workstation (part of office refurbishment) (3 months)	Group interviews and one key informant interview	Three months after workstation installation	Content analysis	Participatory
Hadgraft et al., 2017 [33]	Australia	21 (57%) interviews; 7 (86%) focus groups	136	Government; desk-based, mix of employees and team leaders	Multi-component: individual, organisational, environmental (sit-stand workstation) (12 months)	Semi-structured interviews and two focus groups	Between one and four months after conclusion of the intervention	Thematic analysis	Mixed
Kling et al., 2016 [51]	USA	17 (76%)	18	University; white collar workers	Walking meetings (2 weeks)	Focus groups	After conclusion of the intervention (week 3)	Inductive thematic analysis	Researcher
Leavy et al., 2016 [36]	Australia	Focus groups: 17 (88%); interviews: 12^a^ (67%)	18	Non-government and university; employees and their managers	Sit-stand workstation & instructions by physiotherapist, brief educational intervention (4 weeks)	Semi-structured interviews and three focus groups	Two weeks after conclusion of the intervention	Inductive coding	Researcher
Mackenzie et al., 2015 [34]	UK	17 (88%)	317	University; desk-based	Participatory, low cost multi-level intervention approach (4 weeks)	Open-ended questions	One week after conclusion of the intervention	Thematic analysis	Participatory
Naug et al., 2016 [52]	Australia	n for qualitative unknown.	33	Transport; bus drivers	Group education sessions (3 × 1 h sessions fortnightly over 6 weeks & 1 session at 12 weeks) & pedometer	Group sessions	During education sessions	No analysis	Researcher
Neuhaus et al., 2014 [53]	Australia	Multiple samples: workstation pilot: 5 (60%); control peer group: 7 (86%); Comcare: 18 (gender NR); Stand Up UQ: 13 (gender NR)	Workstation pilot: 5; control peer group: 7; Comcare: 21; Stand Up UQ: 16	University; government workers; desk-based	Pilot testing different aspects of a multi-component intervention (individual, organisational, environmental [sit-stand workstation]). Different samples exposed to various aspects of intervention	Semi-structured interview; telephone feedback	Various	Unclear	Mixed
Such et al., 2017 [26]	UK	13 (46%)	35	University and public sector; desk-based workers (various roles)	Pedometer intervention with individual-level strategies (awareness raising and counselling) (4 weeks)	In-depth interviews	After conclusion of the intervention	Thematic analysis	Researcher
Taylor et al., 2013 [54]	USA	35 (83%)	82	Court reporting, health, education, law; various roles (sitting ≥5 h/day)	Light-mod physical activity routines (15 min/day) led by trained staff at the worksite (6 month duration at 3 worksites; 12 month duration at two worksites)	Open-ended questions	At 6 months (all worksites) and 12 months (worksites with 12-month interventions)	Content analysis	Researcher
Torbeyns et al., 2017 [55]	Belgium	19 (89%)	19	Human resources company; desk-based	Bike desks & email feedback every 4 weeks (5 months)	Open-ended questions	After conclusion of the intervention	Thematic analysis	Researcher
Tudor-Locke et al., 2014 [56]	USA	12 (gender NR)	21	Health insurance; administrative	Shared treadmill desks (3 or 6 months)	Focus groups	After conclusion of the intervention	Unclear	Researcher
Combined non-intervention/intervention studies									
Ahtinen et al., 2016 [23]	Finland	Pre-intervention: 15 (73%); post-intervention: 14 (71%)	14	University; researchers, teachers, coordinators and secretaries	Mobile-mediated walking meetings	Pre-intervention: group discussions while walking; post-intervention: field tests of intervention	Pre-intervention and immediately post-intervention	Content analysis	Researcher
Cole et al., 2015 [57]	UK	Pre-intervention: 7 (14%); post-intervention: 8 (gender NR).	13	Software engineering; employees and managers	Mobile phone application to record activity (2 weeks)	Semi-structured interviews (pre-intervention); open-ended questionnaire (post-intervention)	Prior to, and after conclusion of the intervention.	Thematic analysis	Researcher

Notes: *N/A* Not applicable, *NR* Not reported

^a^Non-participants also interviewed

The majority (88%, 28/32) of studies involved predominately office or desk-based workers. Study participants were drawn from the university sector (*n* = 14), private/non-government organisations (*n* = 13), and government/public sector and emergency services (*n* = 8) (some studies involved multiple sectors). Two studies involved bus drivers, while two involved emergency services workers with operational and non-operational duties. Most studies were conducted in Australia (*n* = 14), USA (*n* = 6) or the United Kingdom (*n* = 6). Sample sizes ranged from five [20] through to 71 participants [21], with a total of 804 participants across all studies. Data collection methods included focus groups/group interviews (*n* = 13), semi-structured or in-depth interviews (*n* = 8), a combination of interviews and focus groups/group interviews (*n* = 6), open-ended survey questions (*n* = 4) or contextual inquiry (*n* = 1). Analysis methods used included thematic analysis (*n* = 13), content analysis (*n* = 5) or grounded theory (*n* = 2), while analysis methods in four studies were inadequately reported or unclear. All of the studies were published between 2008 and 2017.

### Themes of non-intervention studies

#### Reflections on sitting and health effects

Desk-based workers reflected that they spent most of their workday sitting and were interested in the opportunity to reduce workplace sitting. However, there was generally limited knowledge of what amount of sitting time was appropriate to avoid adverse health outcomes, or how often sitting should be broken up during the workday. Although too much sitting was identified as a potential health risk factor, health effects attributable to sitting were generally related to musculoskeletal problems (e.g. sore neck, back) rather than longer-term chronic diseases. Not all workers had a clear understanding of the difference between too much sitting, and physical inactivity (i.e. not meeting recommended guidelines for physical activity, independent of the amount of sitting time accumulated across the day).

#### Expected and experienced benefits of sitting less

The opportunity to sit less at work was perceived to have a number of potential benefits. These included work-related benefits, such as improved productivity through shorter, standing meetings, and gaining new insights or perspectives when walking. Potential health and well-being benefits noted of taking more breaks or walking more included feeling more refreshed, and “*giving [the] brain a little bit of a break”* [12]. In two studies [22, 23], walking meetings or walking clubs were perceived to have social benefits by bringing co-workers closer together.

#### Barriers to reducing sitting

A number of factors were perceived to act as barriers to the ability to reduce workplace sitting time. In line with the ecological model of sedentary behaviour [24, 25], these were grouped under individual-level (e.g. personal preferences, health), work-related (e.g. work load), environmental (e.g. physical office layout), organisational and social-level factors. (e.g. social support). A summary of this information is presented below, while a more detailed list of barriers (and facilitators) to reducing workplace sitting, across intervention and non-intervention studies, is presented in Table 3 with accompanying quotes.

**Table 3:** Barriers and facilitators to reducing workplace sitting across non-intervention and intervention studies

	Non-intervention studies	Intervention studies	Quotes
Individual Level	Barriers	Barriers	
	- Sitting as a long-term habit – hard to change - Individual choice to remain sedentary - Being tired or standing perceived to be tiring/uncomfortable - Concern that reducing sitting equates to “standing all day” - Need to see a personal benefit (e.g. health benefit) - Competing priorities affecting behavioural change	- Habit/forgetting to change posture - Not being aware/able to estimate how much time is spent sitting - Individual preference in sitting/standing for different work tasks - Standing contributed to musculoskeletal discomfort. - Difficult concentrating when standing - Needing further instruction on how to engage with strategies - Need appropriate footwear for standing - Individual frustrations with strategies (see Strategy-specific barriers and facilitators)	Habit:*“ ... I have the intention, but I forget to get up ...”* Employee, intervention study [20] Individual choice: *“I’ve gone from a standing up for 10 hours a day job, so I enjoy the sitting”* Employee, non-intervention study [48]
	Facilitators	Facilitators	
	- Individual motivation or commitment - Feeling personal benefits from reducing sitting	- Individual motivation or personal challenge - Awareness of amount of time spent sitting - Sitting less becoming habitual - Experiencing health benefits from sitting less (such as reduced tiredness, greater concentration or alleviating musculoskeletal problems) - Perceived improvement in concentration/productivity - Having the choice and flexibility to change posture	Individual motivation: *“I used to make a conscious effort to get up out of my desk, talk to people, interact. It comes down to the individual. It’s how you manage your workload on a daily basis.”* Employee, non-intervention study [28]
Work-related	Barriers	Barriers	
	- Work requires the use of a computer (and seated posture) - Work has become more sedentary due to increasing use of technology - Some roles more sedentary than others - Employee/manager perception that taking breaks interrupts work flow/affects productivity - Perception that cognitive work requires people to be sitting down - Caught up in work – not noticing prolonged sitting - Home working and flexi-time strategies lead to more sitting - Shift work as a barrier to being active	- Screen based work - Some job roles not considered appropriate to perform standing (e.g. receptionist) - Some tasks difficult to perform standing - Work load and time pressures limit ability to take breaks or engage with strategies considered an “interruption” to work tasks - Perception that taking more breaks away from workstation equals not meeting demands of the job - Concern about privacy of work when standing	Computer-based work: *“Today we sit down in front of that computer screen regularly for a good portion of a day to do training, to do reports, research whatever it may be, and so the number of hours your ass is in a chair has increased.”* Employee, non-intervention study [21] Sitting breaks as interruption to work: *“So much of my work relies on being sat at a computer which is unable to be addressed in a zero cost intervention”.* Employee, intervention study [34]
	Facilitators	Facilitators	
	- Job tasks able to or required to be performed away from the desk (e.g. managerial duties) - Flexibility when breaks can be taken - Perceiving standing/moving to assist with tasks	- Having work tasks that could be performed walking - Flexibility with time (e.g. when breaks can be taken)	Job tasks: *“We from time to time need to check things in the filing room so we need to get up and go there, but yeah, it*’*s maybe breaking up the job a bit more too. If we had, I guess, other tasks that involved getting up for a period of time that would probably help as well.”* Employee, non-intervention study [12]
Social or organisational	Barriers	Barriers	
	- Not wanting to stand out - Concern about disturbing co-workers - Concern that standing or moving perceived as not working by colleagues and managers - Leaders not convinced about benefits of reducing sedentary behaviour - Workplace culture not supportive of initiatives to reduce sitting - Financial investment associated with sit-stand workstations - Culture encourages siloing and use of email. - Cultural issues: “Asian culture” perceives standing to be aggressive - OHS concerns relating to sit-stand workstations, physical activity, stairwell accessibility - OHS focus on treating issues rather than preventive health approach	- Concern colleagues perceive behaviour to be unusual - Concern about disturbing co-workers - Concern that colleagues/managers perceive standing or moving to be unproductive - Supervisor belief that sitting less reduces productivity - Not all levels of management supportive of intervention - Walking during breaks: less time for social interaction	Social norms: *“So for me there are some hidden pressures, it’s not perceived to be good to be seen walking around unless you’ve got piece of A4 paper in your hand.”* Employee, non-intervention study [45] Disturbing co-workers: *“When I’m on the phone standing up I feel a little bit conscious because I feel like I’m shouting out across everyone and I’m sort of distracting people next to me.”* Employee, intervention study [29]
	Facilitators	Facilitators	
	- Workplace culture where sitting less is the norm - Workplace culture supports regular short breaks - Group activities for motivation - Top down (manager, leadership) support and encouragement for sitting less, including permission for change - Having a workplace champion/role model who can motivate others and model behaviour - Organisation interest, investment and commitment in reducing sitting time - Wellbeing committee that meets regularly to discuss raising awareness and strategies - Support from OHS personnel - Having a reason to go and visit colleagues	- More supportive social norms for reducing sitting after intervention. - Involvement of co-workers in strategies helped to normalise standing/moving more - Managers providing permission - Management leading by example - Workplace champion driving change	Management permission: *As soon as managers say,* “*If you want to stand, feel free to,*” *you can guarantee it there*’*ll be people immediately that will stand because managers have given them that permission to do it and therefore they*’*ve got the permission from everyone else to do it.* Employee, intervention study [33]
Environmental	Barriers	Barriers	
	- Most furniture designed for sitting - Ergonomic issues with standing arrangements - Lack of common spaces away from desk - Stairs difficult to access/ - Lifts more convenient than stairs - Close proximity to co-workers – potential for disturbing others - Communal facilities close to desks - Lack of supportive facilities for activity (e.g. clothes iron) - Weather (heat) as a potential barrier to walking	- Activity-permissive workstations: issues with design (see strategy-specific barriers) - Open plan office: distractions, privacy issues - Weather (cold, rain) as barrier to walking	Furniture designed for sitting: “*You really need higher tables to take notes or to take a look at your papers, you need to have these facilities otherwise people will sit if they have the opportunity*” Executive, non-intervention study [13]
	Facilitators	Facilitators	
	- Provision of sit-stand workstations - Spaces within the building where people can go to take a break - Communal facilities (e.g. printers) located away from individual desks - Nice routes/nearby parkland for walking - Good weather to encourage walking	- Interesting/safe routes for walking around the office	Weather: “*When the weather’s good, I go for a walk.”* Employee, non-intervention study [57]
Strategy-specific barriers and facilitators		Barriers	
		- Intervention emails: not read due to email overload - Activity-permissive workstations (e.g. sit-stand workstations and treadmill desks): unstable surface area, insufficient surface space to work, difficult to adjust monitor distance/workstation height (particularly if manual); inconvenient if only available at certain times of the day (e.g. hot desk arrangements); social hierarchies affected by height differential - Treadmill desks only: difficult to set up and noisy - Walking meetings: too many people in meetings - Lumoback activity tracker: comfort, difficult to wear with clothing, set up or syncing issues - Computer prompts: disruption to work flow	Sit-stand workstation design: *“I thought it [the sit-stand workstation] was a really poor design. Just the way it bounced about and the screen kept moving and cords getting in the way and all this.”* Employee, intervention study [31]
		Facilitators	
		- Sit-stand workstations: allow work to continue while standing - Using prompts or triggers for activity-permissive workstation use (e.g. leaving desks in up position) - Activity trackers: allow monitoring of behaviour - Anti-fatigue mats to accompany sit-stand workstations	

Barriers and facilitators to reducing workplace

At the individual-level, sitting was perceived to be a habitual behaviour that was difficult to overcome, particularly when participants did not perceive a personal benefit to doing so (e.g. immediate health benefits). Some participants also perceived reducing sitting to be an individual choice whereby some workers were more motivated to take breaks than others.

Most participants reported work-related barriers to reducing sitting. As predominately computer-based workers, participants perceived that sitting was an inevitable part of their job. Similarly, some employees and team leaders suggested that taking more frequent breaks from sitting could reduce productivity. Opportunities to move away from workstations depended largely on the job role, such as whether participants had people management responsibilities and the extent of task variation and discretion/control in workload planning.

The social environment, including norms around behaviour, was perceived to be a key influence on workplace activity. Participants were concerned that behaviours such as standing in meetings or taking more regular breaks would be considered as “weird” by co-workers, as going against accepted norms, or as not making a full contribution to the team. There was also a concern that standing or moving could disturb their co-workers.

*“If you’re at any meeting, the norm is to sit there and if you do anything different from that, you immediately stand out and you don’t necessarily stand out in a good light; you’re a bit of a rebel.”* Employee [26]

Workplace cultures that did not support, or actively encourage, initiatives to reduce sitting time (such as standing or walking meetings) was a perceived barrier. Cultures that associated productivity with being at one’s desk were seen to promote sitting and discourage movement. From the perspective of some senior leaders interviewed, there was also a need to ensure that sedentary behaviour interventions (such as sit-stand desks) would be a good financial investment, given associated costs and competing workplace priorities. In a study conducted in Singapore, it was noted that societal cultural factors were a barrier to reducing sitting, with standing perceived to be “*aggressive, very domineering*!” [27].

At the environmental-level, the main barrier to reducing sitting was the predominance of furniture designed for sitting. Incidental activity during the day was also reported to be constrained by the environment, such as the inability to access stairs or outside locations or having insufficient facilities that would provide opportunities for breaks or to support activity.

#### Facilitators for reducing sitting

Perceived facilitators for reducing sitting time were commonly the flipside of reported barriers. At the individual-level, this included perceiving a personal benefit from reducing sitting and being motivated to change behaviour. At the work-level, jobs or tasks able to be performed away from individual computers/workstations (e.g. people leader roles, collaborative tasks) were considered to facilitate sitting less. Participants perceived that demonstrated organisational commitment and support, such as providing resources for strategies or interventions, and encouraging shifts in cultural norms, would assist with behavioural change. Employees often thought that a top down approach was necessary and noted the importance of management permission for staff to stand in meetings or take breaks. Interestingly, managers were not always perceptive of their integral role in behaviour change. Workplace champions or role models were suggested as a potential way to motivate staff and promote an activity-friendly environment. Environmental-level facilitators included pleasant outdoor surroundings and nice weather for walking, spaces within the building that could be used during breaks, and having communal equipment (such as printers, bins) located away from individual workstations. Sit-stand workstations were also suggested as a potential environmental modification that could facilitate reductions in sitting time.

#### Suggested interventions or strategies

When prompted, participants suggested a range of different interventions/strategies that could assist to reduce workplace sitting (see Additional file 2). Most commonly reported were environmental modifications (such as sit-stand workstations or standing meeting rooms), or educational/awareness initiatives highlighting the negative consequences of prolonged workplace sitting and suggesting tips to break up sitting time.

“*For example a measuring campaign can help to confront people with how long they really sit*…*people may react like* ‘*Oh, I just stayed seated for two hours without any movement*’*!*” Manager [13]

With environmental modifications, it was recognised that these were not necessarily sufficient by themselves and needed appropriate guidance and organisational support to be effective.

Low-cost strategies suggested (with some already being used) included walking during breaks, communicating face-to-face with colleagues (rather than emailing), walking to communal printers and bins, and in one workplace, utilising a walking club. These strategies were generally perceived as acceptable and feasible and had the potential to enhance relationships with co-workers. To address the habitual nature of sitting, computer prompts or alarms were suggested, to remind people to take breaks in their workplace sitting. However, there were some mixed feelings about whether forced breaks and the interruption to work flow would be tolerable.

*“The more structure they add to it, the more it becomes another task that has to be done. So even though the bell’s a really good idea, the fact that the bell rings again and ‘Oh god, here we go’.”* Employee [28]

Some participants also suggested strategies designed to increase physical activity, including promoting active travel, having a gym onsite, and running lunchtime exercise programs.

### Themes associated with intervention studies

The main themes identified in relation to intervention studies were motivation for intervention participation, intervention/strategy benefits, barriers to reducing sitting, facilitators for reducing sitting, and acceptability and suggested improvements for intervention strategies. Similarities and differences in findings to the non-intervention studies are discussed where relevant.

#### Motivation for intervention participation

Some studies reported on participants’ motivation for participating in the intervention. Reasons put forward included: perceiving potential health benefits from reducing sitting, novelty or curiosity (particularly around sit-stand workstations), competition with colleagues, or encouragement from colleagues or managers.

*“I wanted to know that I wasn*’*t putting strain on my cardiovascular system and arteries by sitting 8 hours at a time and I just wanted to see if it had a difference to my energy levels and my problems with my back.”* Employee [29]

Some managers/leaders noted that they were motivated to try health initiatives out of a duty of care to their employees, while some participants were motivated because a sedentary behaviour intervention was perceived to be more feasible and less challenging than structured exercise.

#### Intervention/strategy benefits

Participants reported a range of benefits from their intervention experience. These included improved knowledge and awareness — both in relation to the amount of sitting accumulated and evidence relating to the health risks of high amounts of sitting time. Benefits to physical health and psychological and emotional wellbeing were frequently noted. Common experiences across populations were that reducing sitting time had led to less fatigue, improved alertness and concentration, reduced neck and back pain, relief of stress and improved coping capacity.

However, benefits were not universally perceived across the studies — a small number of participants in some studies reported negative experiences, including musculoskeletal issues when standing [20, 29–32].

Participants also perceived that there had been flow-on effects for their work. Specifically, heightened alertness was perceived to improve work performance, while improved social interactions were considered to have been beneficial for issue resolution. One team leader felt improved connectedness with their staff [33]. In the short-term, productivity gains were not considered to be particularly large. However, from an organisational perspective, it was suggested that improved staff health and well-being could have longer-term benefits to productivity.

Positive changes to workplace culture and social norms were also reported. In particular, it was perceived that intervention strategies (such as standing meetings) became more accepted within organisations, with one participant noting that the intervention had “*strengthened/increased the culture of working in more flexible and creative ways*” [34].

However, there was also a perception that this cultural change may diminish over time and the extent of these changes appeared to vary across studies.

Some participants perceived unexpected or unintended benefits from the interventions. These included flow on effects to other health behaviours, such as smoking and eating [35], or a general “*awakening*” prompting health changes [33]. Some participants also reported that they stood more or did more activity outside of work hours as a result of the intervention.

#### Variation in participant experience of sitting reduction interventions

Within individual studies there was variation in how participants experienced the same interventions. Some of the factors that appeared to contribute to these differences in experience included the level of support received from managers/team leaders and colleagues and the subsequent extent of organisational cultural and individual behaviour change experienced. Individual motivation in changing behaviour was also suggested to have played a role.

In terms of strategy use, there was also individual variation in how participants engaged with the intervention, including their patterns of use. With sit-stand workstations, participants variously described using time or task-based prompts to determine when to stand or sit, while others relied on health indicators—such as feeling tired or sore—to change. Other participants reported not having any particular drivers of when they sat or stood.

Participants also differed in the extent to which they found particular intervention components to be helpful. For example, while some liked strategies that enabled them to track their behaviour, others were less interested.

#### Barriers to reducing sitting – Intervention studies

Similar barriers to reducing sitting were identified as within the non-intervention studies, characterised at the individual, work-related, social/organisational and environmental level. A more complete summary is provided in Table 3.

At the individual-level, health concerns, such as musculoskeletal issues when using sit-stand or treadmill desks, acted as a barrier to reducing sitting for a minority. In one study however, this was noted to be only short-term discomfort [30]. A few participants also raised requiring different footwear to stand and move more at work.

Time pressures and the specific work tasks and job roles (e.g. receptionist) participants had to perform also acted as barriers to engaging with intervention strategies (for example, some tasks were considered difficult to perform standing and time pressures limited the ability to take walking breaks).

Similar to non-intervention studies, participants suggested that feeling self-conscious of co-workers’ perceptions of their behaviour was a barrier to sitting less and standing/moving more. Again, there was a concern that sitting less would be considered less productive, something that was raised by one manager as a concern [36]. Without management support some found it difficult to stand more during the workday.

The outside environment was sometimes raised as a barrier to strategies involving walking, specifically the harshness of the weather during colder months [37]. Even with interventions that targeted the internal physical environment, such as implementation of activity-permissive workstations, design issues were raised as a barrier. These included: unstable surface areas on workstations, insufficient space to work, and difficulties adjusting the workstation setup to meet ergonomic requirements [20, 29–33]. Other barriers to reducing sitting specifically related to intervention strategies are summarised in Table 3.

#### Facilitators to reducing sitting – Intervention studies

Similar to non-intervention studies, individual-level facilitators of reducing sitting during an intervention included perceiving health-related benefits from sitting less, or the personal challenge involved in meeting strategy goals and beating previous targets.

Workplace cultures or social norms that were supportive were considered to facilitate strategy use. In particular, other colleagues participating in the intervention appeared to help normalise standing or moving more and challenge existing behavioural norms. Team leader/manager support was considered important for making changes acceptable, while a workplace champion played a key role in motivating participants in one study [38].

At the environmental level, interesting and safe walking routes were perceived to facilitate walking meetings [23]. Although barriers existed in their design (as noted above), the environmental modification of installing activity-permissive workstations was generally considered a key facilitator, as they assisted with “*normalising standing within the workplace*” [33] and provided a way for workers to perform their work without interruptions.

Strategy-specific facilitators included educational/information material that described the health benefits of performing strategies. Activity trackers and similar supports were also considered valuable for assisting participants to monitor their progress and understand how much time they spent sitting.

#### Acceptability and suggested improvements for intervention strategies

Overall, participants generally found interventions to be acceptable. In two of the studies in particular [33, 36], participants expressed disappointment when the sit-stand workstations were removed at the end of the trial. However, despite the often positive feedback, participants had a number of frustrations with, and suggestions for improving, strategies and interventions; these are outlined in Additional file 2. Some of these suggestions involved additional desired strategies whereas others involved modifications to experienced strategies or to the intervention as a whole.

## Discussion

In the last five years there has been increasing interest in understanding the effectiveness and feasibility of interventions aimed at addressing prolonged sitting time in the workplace. In light of the growing number of studies, we aimed to synthesise the available qualitative evidence relating to workers’ perceptions of factors influencing their workplace sitting time, and the feasibility of workplace sitting reduction interventions.

While experienced and perceived benefits of reducing workplace sitting were similar across intervention and non-intervention studies—particularly concerning health and social factors—those benefits reported were generally broader and more extensive following intervention participation. In terms of barriers and facilitators, work and social-related factors were prominent across many studies. Computer-based work is a key driver of the large volumes of time that many office workers spend sitting [6]. With environmental practices encouraging a reduction in paper use and preference for communication to be documented (i.e. in emails) [12], office workers in these studies often had few work-related tasks that could be performed away from the desk. An associated barrier with ‘low-cost’ sitting reduction strategies that promote time away from the desk (e.g. walking to visit co-workers, more regular trips to the kitchen/bathroom), is that they can be viewed by employees and leaders as interrupting and reducing the time available for productive, computer-based work. These perceptions are reinforced by social norms that discourage standing and moving unless there is an agreed reason for doing so. In addition, while these strategies are helpful for encouraging postural breaks, they are unlikely to lead to large reductions in workplace sitting time, relative to interventions such as activity-permissive workstations [9, 39].

Particularly in the non-intervention studies, the concern about managers and co-workers’ perceptions of their behaviour was considered a strong barrier to reducing sitting time. These normative beliefs about ‘appropriate’ office behaviour were identified by workers across multiple studies within this review, across different countries. In particular, a participant in one study conducted in Singapore suggested these cultural norms might be even stronger in ‘Asian culture’, where standing is perceived to be “*aggressive*” [27], rather than just out of the ordinary. In contrast, a recent study in Sweden did not identify cultural or social norms to be a barrier to reducing workplace sitting [40]. The studies in this review were conducted predominately in Australia, the USA and United Kingdom. Further research is needed to understand whether these social norms do differ cross-culturally, particularly in countries where sit-stand workstations are standard office equipment.

Although social norms promoting sitting as the default were viewed as a significant barrier to reducing sitting, encouragingly, these appeared to be amenable to change during an intervention. This was particularly the case when a critical mass of participants was achieved, which created a sense of social cohesion and challenged previous sitting norms. However, peer social support — while seemingly necessary — may not be a sufficient driver of workplace cultural change. Management or team leader engagement and approval was considered crucial for workers to feel able to make changes to their workplace activity. In one study, managers appeared to underestimate the extent to which their approval or endorsement was needed [13], highlighting the need for future interventions to focus on developing this support and ensuring it is communicated to staff.

The intervention studies covered a range of different strategies for reducing workplace sitting time, including activity-permissive workstations, low-cost and organisational support strategies and walking meetings. Participants in the non-intervention studies were also able to suggest many potential strategies when prompted; often those that have been trialled in formal intervention studies. Considering the work-related barriers to reducing workplace sitting (i.e. work pressures and productivity concerns), activity-permissive workstations — particularly sit-stand workstations — were highly valued as they allowed computer/desk-based tasks to continue uninterrupted. While some strategy-specific barriers and facilitators were identified (such as issues with the design of particular sit-stand workstation models), overall, many commonalities existed across studies. This suggests that the social, work-related and physical environment within which ‘sit less’ strategies are implemented is likely to be a significant determinant of the ease of changing behaviour, emphasising the importance of focusing on addressing these multiple behavioural influences during intervention design [41, 42]. Lending support for this approach is the evidence that the greatest reductions in workplace sitting time are observed following multi-component interventions [9].

Although the majority of studies in this review involved desk-based (white collar/ professional) workers, technological advances mean that an increasing number of occupations may now be exposed to the hazards of sedentary work. In one of the few studies involving non-desk based workers [21], firefighters noted that previously hands on training exercises were now simulated, reducing workplace activity. There is a need for research exploring the effectiveness and feasibility of strategies to reduce workplace sitting in a more diverse range of industry sectors, to determine whether the barriers and facilitators — and thus most feasible intervention approaches — may differ.

As noted above, the studies in this review were relatively limited in their cultural diversity. Only one study was identified from Asia (Singapore), with no studies from South America or Africa. The restriction of studies to English-language papers may have contributed to this bias. As cultural differences in non-Western countries may be an important influence on the acceptability and feasibility of addressing workplace sitting [27], research from a broader range of countries is needed to inform future intervention work. The similarity of themes (and quotes) from studies conducted in Australia, the USA and the UK suggests that further research in these localities should be prioritised towards addressing different industry sectors (e.g. manufacturing and transport industries; contact/call centres) or involve novel intervention strategies beyond activity-permissive workstations.

When considering the sustainability and research translation potential of the interventions studied, it is worth noting that only five studies incorporated a participatory approach to intervention development, while the majority (17/22) were researcher-led. It is of interest to understand whether fewer or different barriers to change exist when workplaces are involved in the design process and potentially have greater investment in and ownership of the intervention. Within the studies reviewed, there was recognition that there is no ‘one size fits all’ path to behaviour change, and participants’ experiences and perceptions of the same intervention can differ. Rigid protocols that specify when workers should stand and sit, and which strategies they should use, should therefore be avoided in favour of an intervention design that can accommodate individual and team-level differences in terms of preferences, abilities, job tasks and work flow. Examples of such participatory approaches to reducing workplace sitting are now being trialled and evaluated (e.g. [43]).

Nearly all (29/32) of the studies involved desk or office-based workers, and a large proportion were conducted in university settings (14/32). In addition, less than half (15/32) included perspectives from supervisors, managers, or other relevant stakeholders (e.g. occupational health and safety practitioners). This review therefore predominately reflects the perspectives of workers, not those involved in planning and funding health and wellbeing initiatives in the workplace. While this was predominately the aim of this study, it does suggest a research gap for future studies and reviews to address. Qualitative research was also generally conducted immediately following the conclusion of intervention delivery, limiting potential understanding of factors that might influence the sustainability of behavioural change or the acceptability of strategies/interventions over time.

In the context of the body of research that has been accumulating around the feasibility and acceptability of reducing workplace sitting time, it is intended that this evidence summary will be informative for the design of future interventions. Table 4 summaries the main implications of the findings for researchers and practitioners. A strength was the breadth of this review, which included qualitative studies associated with interventions, and also those exploring workers’ perceptions in the absence of interventions. The use of multiple databases also facilitated the breadth of the review. As noted by the publication dates of included studies (all published after 2007 and 94% published in the last five years), this is a rapidly growing field of interest. With growing interest from government and other stakeholder groups [6, 44] in addressing high levels of workplace sitting time, it is important that the design of intervention strategies continues to be informed by up-to-date research.

**Table 4:** Summary of implications for researchers, practitioners and workplaces

*Implications for intervention design and implementation:* - Approaches to workplace sitting reduction should aim to address the multiple levels of influence on behaviour, including individual, work-related, social and environmental. - The importance of workplace culture and social norms as influences on workplace behaviour should be addressed explicitly. - Ensure that intervention strategies are tailored to individual team and organisational needs. This may require starting with small changes to encourage cultural shifts in sitting less and moving more (e.g. implementing a ‘standing agenda item’ in meetings to encourage standing). - Emphasise the importance of support and leadership across all organisational levels (particularly senior management level) for intervention messaging and strategies. This may require recruiting workplace champions at multiple levels to promote the program and encourage their co-workers. - Plan for, and address the commonly identified barriers and facilitators to particular elements of sitting reduction approaches (e.g. activity-permissive workstations). In doing so, include ongoing assessment to identify additional barriers as they arise. *Implications for future research directions:* - Assess the feasibility and acceptability of reducing workplace sitting time across a wider variety of cultures, and compare and contrast this to the current evidence base. - Prioritise research assessing barriers/facilitators to reducing workplace sitting in non-desk based workplaces, including the feasibility of a broader range of strategies (i.e. beyond sit-stand workstations). Research within countries where workplace sitting research is more prolific (e.g. Australia, the United States and the United Kingdom) should be especially prioritised towards this research. - Conduct qualitative research at multiple stages of intervention delivery, including with a longer-term follow-up to determine if barriers and facilitators to reducing workplace sitting change over time. - Incorporate perspectives from managers and other decision-making stakeholders when assessing the feasibility and acceptability of workplace sitting reduction approaches. - Assess the feasibility and acceptability of participatory, workplace-led interventions to reduce workplace sitting.	

A limitation of this review was that only peer-reviewed studies published in English were included, which may have excluded potentially relevant studies. We also only included peer-reviewed literature, excluding potentially relevant grey or unpublished material, which may have led to a publication bias. In addition, behavioural research is more typically quantitative in nature, thus these studies may not represent the full scope of workplace sedentary behaviour interventions. As there is no commonly agreed upon appraisal tool for qualitative research [19], we did not exclude any studies based on the quality appraisal and findings are therefore limited by the rigour of the included studies. However, generally the studies with lower overall quality scores (particularly in relation to the analysis of the data) provided fewer distinct and useful insights relative to those with higher quality scores. In line with methods described previously [16], our data extraction process included all data included in the Results section of studies. This included both participants’ quotes and the researchers’ interpretations of the findings. We therefore cannot exclude the possibility that the presentation of findings within individual papers was selective or biased.

## Conclusions

This synthesis of qualitative studies has identified a body of research findings on the perceived barriers and facilitators to moving more and sitting less. However, the studies examined reveal limited diversity in country of origin, culture and industry sector. To progress this field and increase the generalisability of findings, future research should seek to better understand the potential barriers and facilitators to reducing sitting in non-desk based occupations in a broader range of countries. As the research conducted to date has also mostly involved researcher-designed and -led interventions, there is a need to evaluate the effectiveness and feasibility of workplace-driven, participatory approaches to addressing workplace sitting, as this may improve the real world applicability and translation potential of findings.

## Additional files

Additional file 1: Search strategy for PubMed. (DOCX 11 kb)

Additional file 2: Suggested improvements to interventions and strategies. (DOCX 16 kb)

## Abbreviations

CASP: Critical Appraisal Skills Programme
N/A: Not applicable
NR: Not reported
UK: United Kingdom
USA: United States of America
