# Supplementary material for: Perceptions of the acceptability and feasibility of reducing occupational sitting: review and thematic synthesis
Source: Int J Behav Nutr Phys Act. 2018 Sep 18;15:90. doi: 10.1186/s12966-018-0718-9 (PMC6145345; doi:10.1186/s12966-018-0718-9)
Supplement: Supplementary file 1 — Search strategy for PubMed. (DOCX 11 kb) [file 12966_2018_718_MOESM1_ESM.docx]

Additional file 1: Search strategy for PubMed

**Work/workplace terms**

worker* [Title/abstract] OR employe* [Title/abstract] OR executive* [Title/abstract] OR workplace [Title/abstract] OR occupation* [Title/abstract] OR organization* [Title/abstract] OR organisation* [Title/abstract] OR worksite [Title/abstract]

AND

**Sedentary behaviour/sitting terms**

Sit [Title/abstract] OR sitting [Title/abstract] OR sedentary [Title/abstract] OR inactive [Title/abstract] OR inactivity [Title/abstract]

AND

**Qualitative research terms**

qualitative [Title/abstract] OR “focus groups” [Title/abstract] OR “focus group” [Title/abstract] OR interview* [Title/abstract] OR “thematic analysis” [Title/abstract] OR “mixed method” [Title/abstract] OR “mixed methods” [Title/abstract]
