# Supplementary material for: Perceptions of the acceptability and feasibility of reducing occupational sitting: review and thematic synthesis
Source: Int J Behav Nutr Phys Act. 2018 Sep 18;15:90. doi: 10.1186/s12966-018-0718-9 (PMC6145345; doi:10.1186/s12966-018-0718-9)
Supplement: Supplementary file 2 — Suggested improvements to interventions and strategies. (DOCX 16 kb) [file 12966_2018_718_MOESM2_ESM.docx]

Additional file 2: Suggested improvements to interventions and strategies

| **Suggested improvements to interventions and strategies**  General suggestions:   - Intervention should be delivered across a broader cross-section of the workforce (not just selective teams) [30] - Email communication should be limited and relevant to avoid information overload [30, 43] - Management supporting strategies and leading by example [41, 43, 45] - Have “workplace champions” who encourage colleagues [41] - Communicate that strategies are evidence-based [41] - Support from study staff (e.g. checking progress) to continue for a longer duration [38]   Walking meetings   - Routes should not be too complicated, should be safe, and long enough to accommodate meeting length [48] - Guidance on how best to conduct a walking meeting [48]   Activity permissive workstations   - Further advice and guidance on how to use the workstation [20, 36, 37, 40] - Ergonomic assessment to ensure suitable adjustment [37, 40, 43] - Higher partitions to address privacy concerns [43, 47] - Greater stability of the workstation [31] - Increased desk space around the workstation [20, 31] - Greater flexibility in adjusting the workstation (e.g. height, distance from user) [31]   Booster breaks   - Variation in exercise routines [45]   **Suggested additional strategies**   - Sit-stand or standing workstations [30, 31, 41] - Standing height tables in meeting rooms [30] - Wearable activity trackers with real-time feedback [30] - Prompts (posters, software) – to remind workers to take breaks [31, 41, 43] - Walking and/or standing meetings [41] - Lunchtime physical activity initiatives [41] - Encourage use of facilities further away (e.g. toilets, kitchens, bins) [41] - Regular health and wellbeing presentations in the workplace [30] - Workplace challenges or competitions (e.g. step counting) [30, 48] - Exercise facilities in the workplace [42] |
| --- |
